# Organelles in the ointment: improved detection of cryptic mitochondrial reads resolves many unknown sequences in cross-species microbiome analyses

**DOI:** 10.1093/ismeco/ycae114

**Published:** 2024-09-24

**Authors:** Dylan Sonett, Tanya Brown, Johan Bengtsson-Palme, Jacqueline L Padilla-Gamiño, Jesse R Zaneveld

**Affiliations:** Department of Pharmacy, School of Pharmacy, University of Washington, Seattle, WA, United States; University of Washington, Division of Biological Sciences, School of Science, Technology, Engineering, and Mathematics, Bothell, WA, United States; Department of Biology, University of Texas at Tyler, Tyler, TX, United States; Division of Systems and Synthetic Biology, Department of Life Sciences, Chalmers University of Technology, Gothenburg, Sweden; Department of Infectious Diseases, Institute of Biomedicine, Sahlgrenska Academy, University of Gothenburg, Gothenburg, Sweden; Centre for Antibiotic Resistance Research (CARe) at the University of Gothenburg, Gothenburg, Sweden; University of Washington, School of Aquatic and Fisheries Sciences, Seattle, WA, United States; University of Washington, Division of Biological Sciences, School of Science, Technology, Engineering, and Mathematics, Bothell, WA, United States

**Keywords:** microbiome analysis, animal microbiomes, mitochondrial diversity, amplicon sequencing, mitochondria

## Abstract

The genomes of mitochondria and chloroplasts contain ribosomal RNA (rRNA) genes, reflecting their ancestry as free-living bacteria. These organellar rRNAs are often amplified in microbiome studies of animals and plants. If identified, they can be discarded, merely reducing sequencing depth. However, we identify certain high-abundance organeller RNAs not identified by common pipelines, which may compromise statistical analysis of microbiome structure and diversity. We quantified this by reanalyzing 7459 samples from seven 16S rRNA studies, including microbiomes from 927 unique animal genera. We find that under-annotation of cryptic mitochondrial and chloroplast reads affects multiple of these large-scale cross-species microbiome comparisons, and varies between host species, biasing comparisons. We offer a straightforward solution: supplementing existing taxonomies with diverse organelle rRNA sequences. This resolves up to 97% of unique unclassified sequences in some entire studies as mitochondrial (14% averaged across all studies), without increasing false positive annotations in mitochondria-free mock communities. Improved annotation decreases the proportion of unknown sequences by ≥10-fold in 2262 of 7459 samples (30%), spanning five of seven major studies examined. We recommend leveraging organelle sequence diversity to better identify organelle gene sequences in microbiome studies, and provide code, data resources and tutorials that implement this approach.

## Introduction

Endosymbiotic theory has amassed considerable evidence that the ancestors of all animal mitochondria were free-living alpha-proteobacteria, while chloroplasts derive from formerly free-living cyanobacteria. Traces of the evolutionary history of mitochondria and chloroplasts as formerly free-living microbes can be found in organelle genomes. For example, mitochondria encode their own version of the small subunit rRNA gene called the 12S rRNA. Such organellar rRNA genes are often amplified by the same PCR primers used in 16S rRNA studies of the microbiome. For example, 16S rRNA sequencing of human esophagus and breast cancer biopsy samples showed high proportions of host mitochondrial reads [1]. Similarly, in a study of the microbiome of 32 plant species, contamination by plastid rRNA genes accounted for ~20% of reads per species on average, but in certain taxonomic groups, that rose as high as 94% [2]. In these cases, mitochondria or plastids were correctly annotated by taxonomic workflows, and so could be readily removed *in silico*. This reduces sequencing depth—any reads spent sequencing chloroplast or mitochondrial rRNAs do not help detect free-living microbes—but otherwise poses few challenges for microbiome analysis. However, if organelle rRNAs from diverse host taxa are not correctly annotated by typical taxonomic annotation workflows, there is a risk of incorrect biological conclusions unless these cryptic organelle sequences are manually identified and removed by investigators.

Mitochondrial and chloroplast rRNA sequences have in some studies been shown to be misclassified by common workflows - often as “Unclassified” microbes, or bacteria of unknown phylum. For example, in a study of black corals [3], typical methods failed to taxonomically annotate a 12S mitochondrial OTU representing 47% of all quality-filtered reads and present in 70% of samples. This OTU was only identified by manual review and required additional analysis to annotate and remove. In other cases, unusually high proportions of unassigned sequences suggest misclassification of some organelle rRNAs. In a study of the effect of maize root exudates on microbiomes, some categories of samples show up to 21.3% of sequences labeled as “Unassigned” at the domain level, even after putative removal of mitochondrial or chloroplast sequences [4]. Similarly, the most influential sequence cluster contributing to the similarity of two species of cold-water octocorals in genus *Primnoa* was an “Unassigned” sequence [5]. In these cases, it is essential to ensure that diverse mitochondrial rRNA sequences are consistently annotated, so they can be removed prior to microbiome analysis. Instances where highly abundant organelle sequences are not correctly identified by taxonomic annotation tools at best create extra work (e.g. requiring additional ad hoc workflows to fix incorrect annotations), and at worst risk altering biological conclusions about microbiome diversity and structure. Because organelle sequences are often very high abundance, errors arising from failure to identify and remove them may be substantial.

**Figure 1:**
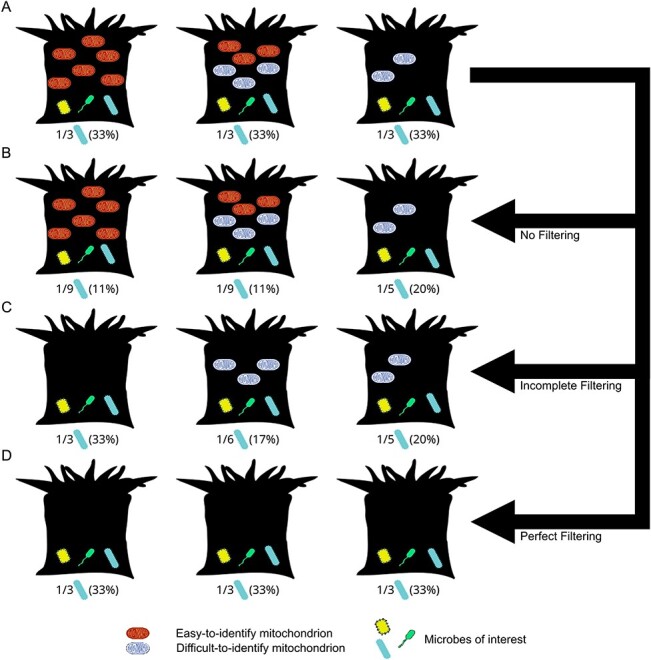
Conceptual diagram illustrating how taxonomically-biased misannotation of mitochondria can distort apparent relative abundances of microbes between animal species. **A.** a set of 3 coral species with identical microbiomes, each of which has a rod-shaped microbe with a true abundance of 33% (1/3 microbes) but with variation in abundance and type of mitochondrial rRNA sequences (light and dark mitochondria). **B.** Analyzing microbiomes without removing mitochondria inflates the relative abundance of the microbe in samples with few mitochondria (3rd column). **C.** Incomplete removal of mitochondrial sequences (dark but not light) distorts relative abundances based on both number and kind of mitochondria. **D.** Perfect filtering of mitochondrial sequences removes mitochondrial abundance as a source of bias in diversity analysis.

The problem of organelle sequence removal is made much more challenging if multiple types of organelle marker gene sequences are present in a study, but each varies in abundance between samples (see example with mitochondria; Fig. 1A). In such cases, taxonomic annotation workflows that annotate *some* but not all organelle sequences are particularly problematic, as they may give investigators a false assurance that all organelle sequences have been annotated. For example, mitochondrial reads from diverse sources in an animal’s diet may be present in animal guts, while diverse microbial eukaryotes (each with unique mitochondria) are commonly found on tropical corals. Because microbiome data are compositional [6–8], failure to remove mitochondrial or chloroplast sequences can distort the apparent relative abundance of bacteria and archaea present in the samples (Fig. 1B). Worse, biased annotation and removal of mitochondrial or chloroplast reads can further distort relative abundances if organelle rRNA sequence diversity causes some mitochondrial sequences to be annotated while others are not (Fig. 1C). A key goal, therefore, is to uniformly identify all organelle-derived rRNA sequences, thus preventing these reads from biasing downstream analyses (Fig. 1D). If mitochondrial or chloroplast contamination is identified and removed in some host species, while not identified or removed from other host species, there is potential to inflate cross-species microbiome differences.

The issue of organelle sequences confounding 16S rRNA gene studies has been addressed by excluding organelle sequences using molecular or *in silico* methods. Several molecular methods for exclusion of organelle SSU rRNA gene sequences have been developed, including peptide-nucleic-acid (PNA) clamps [2] and Crispr-Cas9 cleavage [9]. However, such methods must generally be adapted to each host separately based on the host’s mitochondrial rDNA sequence, which may make their application challenging in cross-species surveys such as the Sponge Microbiome Project [10] and Global Coral Microbiome Project [11]. Additionally, applying such methods adds time and complexity to analyses and cannot be applied retroactively to existing studies without re-amplification and resequencing of the underlying samples. Application of different molecular mitochondrial removal protocols tailored to specific taxonomic groups may also have difficult to quantify effects on the comparability of diverse studies in meta-analysis.

An alternative approach is to identify and filter out organelle rRNA sequences *in silico* using standard taxonomy annotation pipelines such as the naive-Bayesian RDP classifier [12] and alignment-based algorithms such as USEARCH [13] and VSEARCH [14,15]. If this process is accurate and unbiased across categories of samples, then removal of organelle rRNA sequences reduces effective sequencing depth but does not otherwise compromise microbiome analysis. Additionally, because *in silico* methods retain organelle rRNA sequences, they allow for separate analysis of these sometimes valuable data. For example, when rRNA reads deriving from eukaryotic organelles are correctly and consistently annotated, they have been used to provide valuable insights into eukaryotic components of microbiomes, such as studies exploiting plastid rRNA sequences to assess the diversity of eukaryotic phytoplankton [16]. Analysis of such “bycatch” from eukaryotic organelle in microbiome datasets has even led to discoveries of novel and globally distributed clades of eukaryotes, including important expansions of known apicomplexan diversity [17].

Application of *in silico* organelle rRNA identification methods to animal microbiomes typically does identify some mitochondrial 12S rRNA gene sequences. However, the existing literature does not establish whether existing workflows annotate all mitochondrial 12S rRNA sequences, or if additional mitochondrial sequences might be present in samples but under-annotated. In this manuscript we report widespread, severe, and host taxonomy specific underannotation of mitochondrial 12S rRNA sequences in several animal microbiomes when using standard taxonomy resources, and suggest a simple improvement that addresses the issue. Popular workflows typically identify some of the mitochondrially-derived reads in each sample. Surprisingly, however, some of these workflows do not necessarily annotate all, or even most mitochondrial sequences (similar to Fig. 1C). We demonstrate that this issue is taxonomically widespread — it severely affects analyses of the microbiome of reef-building corals, and to a lesser extent those of marine sponges, ants, birds and mammals. We develop an extended set of taxonomic annotations that are supplemented with diverse known mitochondrial 12S rRNA gene sequences, and demonstrate that this extended taxonomy resolves the provenance of the vast majority of “Unassigned” sequences in some studies without causing false positive mitochondrial annotations.

## Materials and methods

### Workflow code

Analyses were conducted using Jupyter notebooks, python scripts, and shell scripts. Major analysis steps are also illustrated in a workflow diagram (See online supplementary material for a colour version of this figure, Fig. S1). Unless noted otherwise, default parameters were used for each analysis step. The QIIME2 bioinformatics platform (qiime2-2023.5) was used for microbiome analysis, except where noted. The full set of code, along with tutorials on using the V4 extended reference taxonomies described here are publicly available on GitHub (https://github.com/zaneveld/organelle_removal).

### Initial generation of the global coral microbiome project dataset

Coral microbiome DNA sequences were selected from samples collected by the Global Coral Microbiome Project (GCMP) as described in Pollock *et al*. [11], but including additional locations outside of Australia in Panama, Saudi Arabia, Columbia, Singapore, and La Réunion that were not described in that manuscript. Importantly, these samples have been sequenced twice: once using Illumina (Illumina, Inc., San Diego, California, USA) MiSeq sequencing, and again using the EMP protocol and Illumina HiSeq sequencing. The samples analysed here were sequenced following the EMP protocol, as this was the larger sample set, and also used standardized methods applied to diverse study systems.

Briefly, samples were collected from water, sediment, and the mucus, tissue, and skeleton of corals from 457 coral colonies, then DNA was extracted using the MoBio Powersoil DNA Isolation Kit (MoBio Laboratories, Carlsbad, California, USA) and processed by the Earth Microbiome Project at the Center for Microbiome Innovation (University of California San Diego, San Diego, California, USA). PCR was run on the V4 region of the 16S rRNA gene using 515f (5’-GTGTGCCAGCMGCCGCGGTAA-3′) and 806r (5’-GGACTACHVGGGTWTCTAAT-3′) primers and sequenced using Illumina HiSeq with 125 bp paired-end reads. Sequences were downloaded from the Earth Microbiome Project via Qiita project ID 10895 (specifically prep id 3439). In Qiita, these sequences were processed using standard EMP workflows: fastq files were demultiplexed using 12 bp Golay codes with the QIIME 1.9.1 split_libraries script (default parameters), trimmed to 100 nt, and then subjected to quality control with Deblur 1.1.0 (default parameters). The “deblur final table” artifact (ID: 59201, now deprecated) was used for initial investigations.

### Initial detection of high numbers of mitochondria annotated as "unassigned" in the GCMP dataset

Many samples in the GCMP dataset were discovered to have high proportions of reads which were labeled "Unassigned" by the QIIME2 feature-classifier plugin [18], using the classify-consensus-vsearch method [14] and the Greengenes 13_8 reference taxonomy. The 1000 highest frequency "Unassigned" sequences were queried with blastn against the nt database (blast_unknowns.py), with the following options: '-max_target_seqs 5', '-max_hsps 1', '-outfmt 6 qseqid sseqid staxids stitle evalue bitscore'.

### Study selection

To determine if under-annotation of mitochondrial reads was widespread across multiple studies, we reanalysed the original sequences from five additional studies in the Qiita database, covering sponges [10], diverse vertebrates [19], humans [20], bovine milk [21], and ants [22]. These studies were selected to represent a range of animal-associated study systems in which we expected mitochondrial sequences to be present. Samples from mockrobiota [15,23–27], a collection of artificially constructed (mock) microbial communities which were known to lack mitochondria, were used as negative controls.

### Construction of extended SILVA and Greengenes databases

Reads were downloaded and extracted from the Metaxa2 [28] custom BLAST database. Mitochondrial reads in this database were themselves curated from Mitozoa (version 2.0, release 10) [29] and SILVA (release 111) [30]. Chloroplast sequences were collected from the Phytoref database [31]. The V4 region of the SILVA 138 taxonomy reference was downloaded from the data resources page of QIIME2 2021.4 (preprocessed with reSCRIPT). The Greengenes 13_8 reference was downloaded from greengenes.microbiome.me, imported into QIIME2, and the V4 region was selected with the q2-feature-classifier plugin. The V4 region was selected due to its use by projects following the Earth Microbiome Project protocol, including the GCMP, where the initial investigation began.

Mitochondria sequences from the Metaxa2 reference database and chloroplast sequences were imported into QIIME2 and similarly limited to the V4 region with reSCRIPT. They were then inserted into the SILVA and Greengenes databases using the q2-feature-table plugin. In all cases, taxonomy strings were reformatted to match SILVA or Greengenes conventions, respectively. This resulted in new custom databases which we refer to as “extended” reference taxonomies (e.g. Greengenes (Extended)).

### Initial data processing and quality control

While Qiita offers the ability to download fully-processed biom tables, the commonly used DADA2 denoising algorithm is not incorporated into available processing pipelines. To apply DADA2, the raw upstream fastq files of each study were downloaded from Qiita and imported into QIIME 22021–4. After demultiplexing (q2-demux emp-single [32] [33]), sequences from Yatsunenko *et al.* were converted to Phred33 from Phred64 by exporting and reimporting into QIIME 2. Each study was separately denoised with DADA2 (q2-dada2) and Deblur (q2-deblur).

### Annotation and benchmarking workflow

Sequences were classified using the QIIME2 feature-classifier [18] plugin with the classify-consensus-vsearch [14] and classify-sklearn [34] methods, with base and extended reference taxonomies. Taxa counts were generated with q2-taxa barplot, after which mitochondria and chloroplasts were filtered from the feature tables (q2-feature-table filter-features). Samples in each table were rarefied to 1000 sequences, after which samples not present in every feature table of each study were discarded to allow for direct comparison of composition and diversity across studies and methods. This rarefaction depth was based on the study design of several of the analysed datasets and was selected to preserve sufficient samples for cross-comparison after organelle removal.

### Testing the effects of Deblur’s SortMeRNA positive filter step

We noticed a substantial difference in annotations of “Unknown” and “Mitochondria” between denoising algorithms (DADA2 vs Deblur) when we explored the effect of supplemented reference taxonomies. In QIIME2, the Deblur plugin uses a “positive filter” step in which SortMeRNA [35] is used to search sequences against a reference 16S rRNA database (Greengenes 13_8 88% OTUs by default), discarding any sequences below 65% identity with 50% coverage to at least one reference sequence. This filtering step is not present when DADA2 is used. We hypothesized that the SortMeRNA positive filter—rather than algorithms themselves—might be responsible for the differences. We tested this using two analyses: first, we ran a version of Deblur in which we disabled the SortMeRNA positive filter by inputting reference sequences matching each query, causing all sequences to avoid the filter. The results of this procedure are labeled “deblur_unfiltered”, in contrast to the default, positively filtered Deblur results, which we refer to as “deblur_filtered”. Second, we added a SortMeRNA filtering step after denoising with DADA2, using SortMeRNA 4 outside of QIIME2 with parameters identical to the implementation in QIIME2 Deblur. This tested whether addition of a positive filtering step would be sufficient to bring the levels of mitochondrial rRNA exclusion for DADA2 in line with that of Deblur. We call the default DADA2 results “dada2_unfiltered”, and those with a SortMeRNA positive filter added “dada2_filtered”. Across these four categories of results (deblur_unfiltered’, deblur_filtered, dada2_unfiltered, and dada2_filtered), we compared the proportion of unassigned reads.

### Testing changes in diversity analysis

Under-annotation of mitochondrial reads has the potential to alter alpha or beta diversity estimates, especially when under-annotation varies between sample categories (e.g. if different host species are being compared). To quantify these effects, we took samples from each study (considered separately) and compared alpha and beta diversity results when comparing the SILVA vs. SILVA (Extended) reference taxonomies. We tested all combinations of denoising algorithm (Deblur vs. DADA2), classifier (VSEARCH vs. naive Bayes), and filtering (presence or absence of a positive SortMeRNA filter). Within these datasets, we selected metadata categories per study for comparison of alpha and beta diversity. These were anatomy ('tissue_compartment') and family-level taxonomy ('family') for Pollock et al., species (“host scientific name”) and sample type (“empo3”) for Thomas et al. life stage (“life_stage”) and environment (“env_biome”) for Yatsunenko et al., “season” and silo (“silo_lot_id”) for Kable *et al*, “genus” and “habitat” for Sanders et al., and “class” and “country” for Song et al. QIIME2’s diversity plugin was used to calculate effect sizes and P-values for Faith’s phylogenetic diversity, observed features, Shannon diversity, Simpson’s evenness, unweighted Unifrac, weighted Unifrac, the Jaccard index, Aitchison distance, and Bray–Curtis dissimilarity across all combinations of previously mentioned variables. Comparisons of alpha diversity were calculated with the Kruskal–Wallis test, while beta diversity differences were calculated with permutational multivariate analysis of variance (PERMANOVA) with 999 999 permutations in order to achieve higher precision *P*-values.

## Results

### High proportions of “unassigned” sequences in coral microbiomes

We used a global survey of coral microbiomes as a case study to investigate purported systematic errors in taxonomic annotation. In theory, common taxonomy annotation pipelines should either annotate all mitochondria-derived reads as such, or make clear in documentation that they cannot annotate mitochondria. We encountered this issue in analyzing data on coral microbiome diversity as part of the GCMP. This analysis collected DNA samples from phylogenetically diverse corals around the world (Supplementary Data Table S1A), and sequenced 16S rRNA gene amplicon libraries from them as part of the broader Earth Microbiome Project. In preliminary taxonomic analysis using QIIME 2 [36], we found that many samples showed extremely high proportions of microbes annotated as “Unassigned” at the domain level (data not shown but replicated in later analysis; see below and Supplementary Data Table S2A), despite other amplicon sequences in the dataset being annotated as mitochondrial.

In principle, these reads of “Unassigned” taxonomy might represent novel diversity or sequencing artifacts. If these truly represented novel domain- or phylum- level diversity, that would be very surprising, given that such novel diversity has not appeared in studies of full-length 16S rRNA sequences from corals [37], despite the identification of coral-specific members of several known phyla. Additionally, since the standardized sequencing methods used in the GCMP were also used in many other studies in the broader Earth Microbiome Project, it would be surprising if such high proportional abundances of “Unassigned” reads were due purely to sequencing artifacts.

A third explanation, which we regarded as by far the most likely one, is that unassigned sequences could represent under-annotated “cryptic” organelle rRNA sequences that were missed by taxonomy annotation, even as other organelle rRNAs were successfully annotated. We found that many of these “Unassigned” reads had strong BLAST hits to known mitochondrial sequences of corals, algae, diatoms and other marine organisms (Supplementary Data Table S3), as well as potential contaminants (e.g. human mitochondrial rRNA). We used BLAST to confirm the identity of the 1000 most abundant sequences in the GCMP dataset annotated as “Unassigned” by VSEARCH using SILVA 138 as the taxonomic reference. Hits to mitochondrial sequences comprised 56% (1283/2296) of total top 5 BLAST hits with e-values below 10^−10^. Yet although some reads that showed high sequence similarity to mitochondria by BLAST were annotated as “mitochondria” by VSEARCH, most were annotated as “Unassigned” at the domain level. This discrepancy between readily identified mitochondrial sequences and misannotated “cryptic” mitochondrial sequences within the same dataset persisted regardless of whether the SILVA or Greengenes database was used, despite substantial sequence similarity of many “Unassigned” sequences to known mitochondrial sequences. Therefore, we chose to explore the generality of this phenomenon, its effects on microbiome analysis, and potential solutions.

### Supplementation of taxonomic references with more diverse organelle rRNA sequences resolves many unknown sequences as mitochondria.

If many “Unassigned” rRNA reads do, in fact, represent mitochondria (e.g. rather than sequencing artifacts or novel diversity), we should expect that adding reference sequences for known mitochondrial rRNAs from diverse hosts should reduce “Unassigned” annotations and increase mitochondrial annotations. Conversely, there is no reason to expect either sequence artifacts or microbes from novel lineages to show any special degree of sequence similarity with animal mitochondria. Thus, if either sequencing error or taxonomic novelty explained “Unassigned” sequences, we should expect little change in sequence annotations when improving the diversity of mitochondrial sequences represented in reference taxonomies.

**Figure 2:**
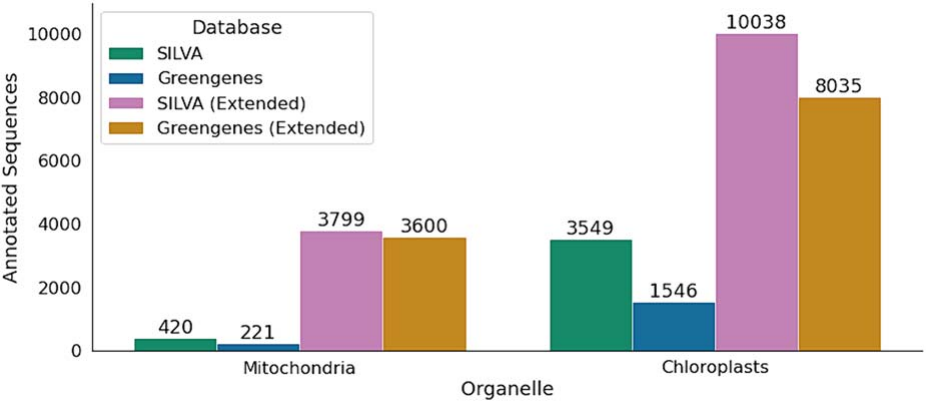
Counts of sequences annotated as mitochondria or chloroplasts in standard or extended reference taxonomies. SILVA refers to the SILVA 138 release; Greengenes to the Greengenes 13_8 release. Extended reference databases incorporate the original SILVA or Greengenes database supplemented with additional organelle sequences from the Metaxa2 database.

We quantified the number of mitochondrial and chloroplast reference sequences found in SILVA version 138 [30,38] and Greengenes 13_8 [39] (Fig. 2, Supplementary Data Table S4). We then collected additional mitochondrial and chloroplast rRNA gene sequences from Metaxa2 [28], and generated extended taxonomic reference databases by integrating them into either SILVA 138 or Greengenes 13_8. This greatly expanded the number of mitochondrial and chloroplast sequences in each reference (Fig. 2). The additional sequences increased the number of mitochondrial sequences in SILVA from 420 to 3799 (approximately 9-fold; Fig. 2), and the number in Greengenes 13_8 from 211 to 3600 (approx. 16-fold). Chloroplast sequence supplementation increased Chloroplast rRNA diversity by 2.8-fold in SILVA 138, or 5-fold for Greengenes 13_8 (Fig. 2). We documented methods for applying the extended taxonomic references within the QIIME2 software package, as well as methods for updating future releases.

We tested how adding these additional mitochondrial reference sequences affected mitochondrial annotation when using different combinations of denoising algorithm (Deblur [40] or DADA2 [23]), base taxonomic references (SILVA [30,38] or Greengenes [39]), and taxonomic classification methods (VSEARCH [14] or naive Bayes [18]). We applied these tests to multiple datasets (Supplementary Data Table S2a-e). These included data from the human microbiome [20] and milk microbiomes [21], as well as multiple cross-species surveys [10,11,19,22] within animal groups (including ants [22], marine corals [11], marine sponges [10], and other diverse vertebrates [19]).

### Effects of extending reference taxonomies differ across studies and animal groups.

**Figure 3:**
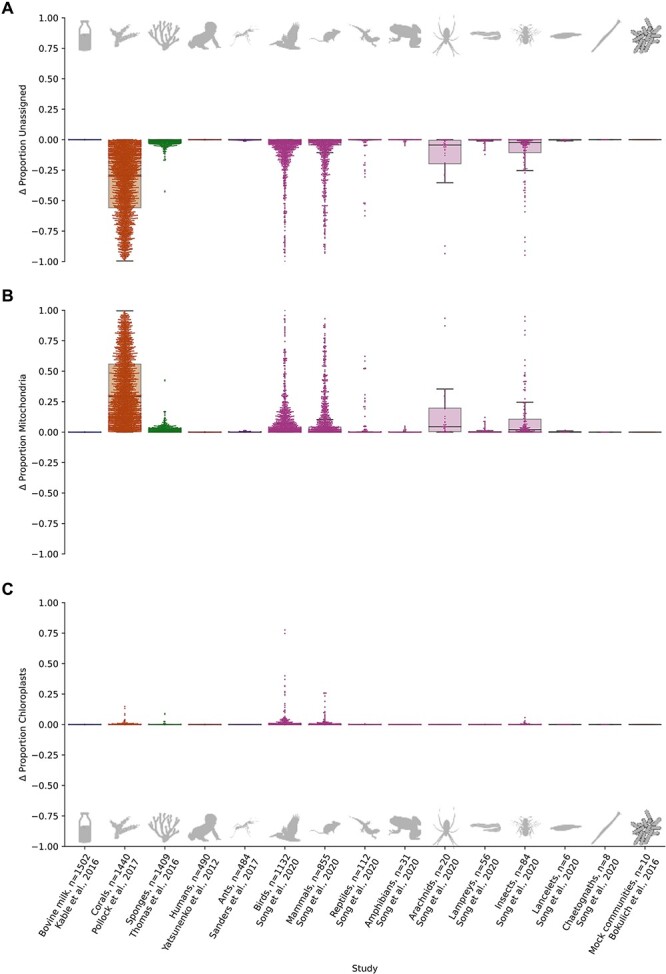
**Supplementation of SILVA with diverse mitochondrial sequences resolves many "unassigned" microbes as mitochondria.** The y-axis shows differences in the apparent relative abundance of unassigned, mitochondrial and chloroplast sequences when 16S rRNA gene sequencing data from several studies (x-axis) were re-annotated using a version of SILVA 138 with additional mitochondrial references (methods). **A.** Difference in the proportion of reads that were unassigned (e.g. “unassigned” at domain level). **B.** Difference in the proportion of reads that were classified as mitochondria. **C.** Difference in the proportion of reads that were classified as chloroplasts. Study labels include the clade studied, author, and number of samples. Annotation with the extended reference taxonomy decreased the proportion of unknown sequences by 10-fold or greater in 2262 of 7459 samples (30%), including representatives from 5 of 7 studies examined (71%), and these decreases were largely matched with proportionate increases in mitochondrial annotations.

Addition of diverse reference mitochondrial sequences had very large effects on analysis of diverse animal groups (Fig. 3) for which proportionally few sequenced genomes are available (e.g. marine corals and sponges), but little effect in several single-species studies of well characterized animal hosts (e.g. in human microbiomes). Importantly, when expanding reference taxonomies decreased “Unassigned” annotations (Fig. 3A), it typically also increased mitochondrial annotations (Fig. 3B), consistent with many “Unassigned” reads representing cryptic organelle sequences, rather than sequencing artifacts or novel diversity. Adding additional chloroplast diversity to taxonomic references also modestly increased chloroplast annotations (Fig. 3C) in studies that included herbivores (e.g. diverse birds and mammals), although these changes were minor compared to shifts in mitochondrial sequence annotation.

**Figure 4:**
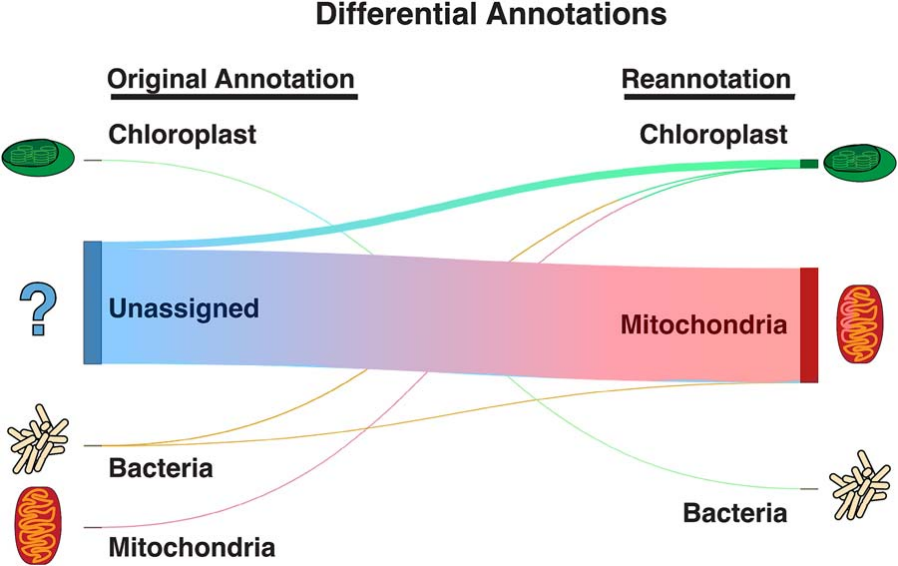
**Taxonomic reclassification using the extended SILVA taxonomy.** Sankey diagram traces show the changes in sequence classification between base (left) and extended (right) SILVA 138 taxonomy at the domain (or organelle) level, weighted by total sequence frequency across all studies (Supplementary Data Table S5). Wider bars indicate reclassification of either many ASVs or a smaller number of ASVs with high frequencies. The most common alterations were of unassigned sequences reassigned to mitochondria (~5.2 million out of 5.6 million total altered annotations, 93.1%), followed by unassigned reads being reassigned to chloroplast (~360 000 / 5.6 million annotations, 6.5%). No unassigned sequences were reannotated as non-organelle bacteria, archaea, or Eukaryota.

Examining differential annotations confirmed that the vast majority of reannotations at or above the class level were formerly “Unassigned” sequences reassigned as mitochondrial (93%) or chloroplast (6.5%) sequences (Fig. 4, Supplementary Data Table S5c). The only other trend notable in these reassignments was that at finer levels of taxonomic resolution some annotations shifted in their specificity (e.g. from species to genus level identification of some Firmicutes; Supplementary Data Table S5g). Notably, independent benchmarks of taxonomic analysis from 16S rRNA data using mock communities have shown overconfident results below the family level [18], suggesting that small shifts towards more conservative annotations in some cases are unlikely to obscure useful biological patterns.

### A positive filter against known 16S rRNA sequences also prevents mitochondrial contamination.

**Figure 5:**
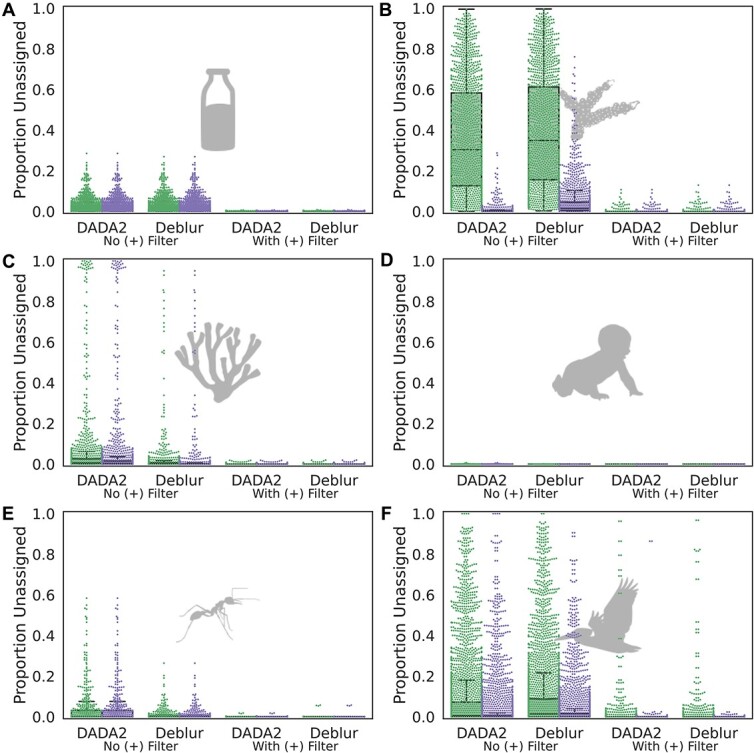
**Positive filtering against known 16S rRNA gene diversity removes many cryptic mitochondrial reads.** To determine the cause of the substantial difference in “unassigned” annotations when using different denoising methods, such as Deblur and DADA2, we separately investigated the methods and the SortMeRNA positive filter generally applied to the QIIME2 implementation of Deblur. Using the filter severely reduced differences in the proportion of unassigned sequences across denoising methods and base vs. extended SILVA reference taxonomies, relative to the unfiltered (unshaded) samples (excepting **D.** human gut samples from Yatsunenko et al. in which samples were extremely well-characterized). **A.** Bovine milk samples from Kable et al. **B.** Coral samples from Pollock et al. **C.** Marine sponge samples from Thomas et al. **D.** Human gut samples from Yatsunenko et al. **E.** Ant gut samples from Sanders et al. **F.** Diverse vertebrate samples (all classes) from Song et al.

The default Deblur pipeline implemented in QIIME 2 includes a “positive filtering” step that excludes sequences below a 65% sequence identity threshold and 50% coverage threshold with the Greengenes 88% OTU reference taxonomy. We tested the effects of this step by either disabling the filter in Deblur or adding it to DADA2 (Fig. 5). We find that this positive filtering step explains most differences between Deblur and DADA2, and eliminates many cryptic mitochondrial reads (Supplementary Information). However, even with a positive filter, the extended taxonomies seem to influence mitochondrial annotations in some samples. For example, even with a positive filter, extended taxonomies detected additional cryptic mitochondrial reads and reduced the number of samples with high levels of Unclassified sequences in the Song et al. dataset of diverse vertebrate microbiomes (see rightmost columns in Fig. 5F).

### Extended mitochondrial reference taxonomies do not promote false positive annotations.

A potential concern about expanding reference taxonomies with extra mitochondrial sequences (some of which are lower in quality than average for Greengenes or SILVA) is that it might lead to false positive annotations of mitochondrial taxonomy. We used two approaches to test for this. First, we annotated the taxonomy of microbial communities of known composition (mock communities [15,23–27]) using either standard or extended taxonomies. Since these mock communities were constructed without mitochondria, we treated any mitochondrial annotations as false positives. However, the extended taxonomies did not increase mitochondrial annotations in these mock communities (Fig. 3B).

We further used shuffled sequences to test the potential that extended taxonomies could increase false positives in which sequencing artifacts were mis-annotated as mitochondria (Supplementary Information, Fig. S2). While the naive Bayes classifier could detect even shuffled mitochondria (presumably based on mononucleotide frequencies), with VSEARCH no false positive mitochondrial annotations were identified in shuffled sequences (Supplementary Data Table S6).

### Under-annotation of mitochondrial reads can influence alpha and beta-diversity.

**Figure 6:**
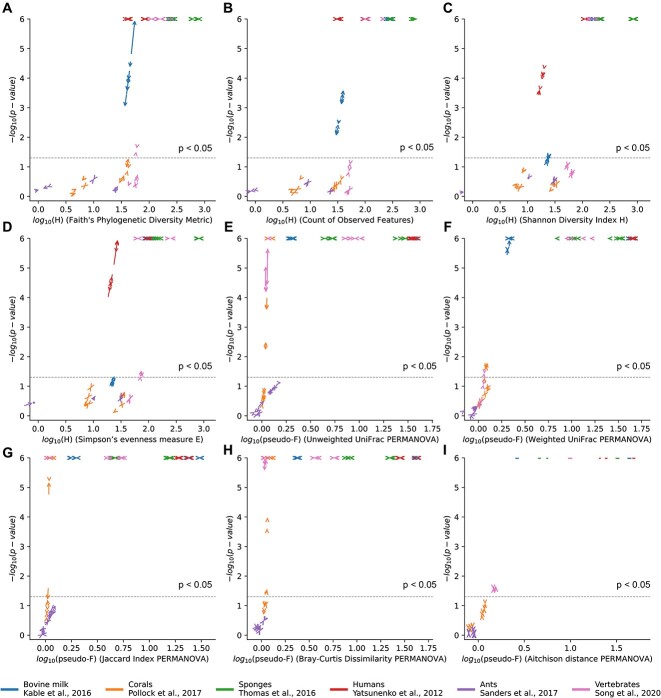
**Change in effect size and significance between base and extended taxonomy references.** Changes in taxonomic classification and filtering can affect both the effect size and significance of Kruskal-Wallis and PERMANOVA tests comparing alpha (**A**, Faith’s phylogenetic diversity; **B**, count of observed features; **C**, Shannon diversity index; **D**, Simpson’s evenness) and beta diversity (**E**, unweighted UniFrac; **F**, weighted UniFrac; **G**, Jaccard index; **H**, bray–Curtis dissimilarity, **I**, Aitchison distance) across metadata categories. Arrow plots show the effect of using the extended taxonomy on study-specific tests (see Supplementary Data Table S7 for a list of metadata categories tested and statistical results). Alpha diversity p-values are capped at 10^−6^ for clarity of visualization, while beta diversity p-values are limited by the PERMANOVA permutations (10 [6]). The dashed line represents p = 0.05. Multiple comparisons may necessitate adjusting this cutoff.

Contamination by organelle rRNAs has the potential to distort alpha and beta diversity comparisons. To investigate this, we reran select alpha and beta diversity analyses for each study after using different mitochondrial removal methods and compared the results (Fig. 6). We compared alpha and beta diversity against two relevant categorical factors per study (e.g. host taxonomy in corals, sponges, and ants; class of vertebrate; milk storage silo; Supplementary Data Table S7). These specific categories were chosen based on the reported results of each study. The results indicate that most differences in comparisons of alpha and beta diversity are more subtle than for taxonomic analysis. We compared differences across categorical variables in each study using four alpha diversity metrics (Faith’s phylogenetic diversity, Fig. 6A; observed features, Fig. 6B; Shannon diversity, Fig. 6C; Simpson’s evenness measure E, Fig. 6D) and five beta diversity measures (Unweighted UniFrac distance, Fig. 6E; Weighted UniFrac distance, Fig. 6F; Jaccard distance, Fig. 6G; Bray–Curtis dissimilarity, Fig. 6H; Aitchison distance, Fig. 6I). Reassuringly, in these large datasets, overall differences in estimated effect size attributable to cryptic organelle sequences were modest, ranging from 0.85-fold to 1.09-fold. Generally these changes in effect size were greater for qualitative, presence-absence based beta diversity measures (e.g. Jaccard distance) than for quantitative ones (Weighted UniFrac or Bray-Curtis).

In several cases changes in effect size and p-value were sufficient to result in crossing the significance threshold of the test (Fig. 6; Supplementary Data Tables S7a-d), potentially affecting biological conclusions. Across all comparisons, the mean absolute shift in p-values by annotation was 0.006, with more comparisons shifted downward in p-value when the extended taxonomy was used. When we separately reran PERMANOVA analysis (1000 permutations per test, 100 replicates per analysis) for beta diversity tests on the GCMP dataset, we found that improvements in p-values from more correct annotation and removal of mitochondrial rRNAs in several cases markedly exceeded replicate-to-replicate variation in PERMANOVA scores. Broadly, the effects of mitochondrial removal on these tests varied greatly across samples and comparisons, likely based on the abundance of mitochondria in the samples and how well mitochondrial rRNAs from particular hosts matched database examples - but in cases where it does matter (e.g. because cryptic mitochondrial sequences are present), these choices can affect biological conclusions. Thus, while critical for accurately showing microbial taxonomy or for separate analysis of all mitochondrial sequences, identification and removal of mitochondrially-derived reads can also somewhat improve the ability to detect biologically interesting trends in host-associated microbiomes.

### Compositional data analysis methods are resistant to under-annotation of mitochondrial reads.

Compositional Data Analysis (CoDA) tools like Analysis of Composition of Microbiomes with Bias Correction (ANCOM-BC) inherently account for the compositional nature of microbiome samples. Because these compositional tools work with log-ratios of microbes, they should not be affected by retention or removal of mitochondria (Methods and Results in Supplementary Information). We confirm that choice of mitochondrial removal method does not affect ANCOM-BC results for non-mitochondrial ASVs (Supplementary Information). Similarly, differences in Aitchison distances (Euclidean distances of unrarefied data transformed with a centered log-ratio) between feature tables filtered with extended or base taxonomic references were minimal compared to other beta-diversity measures (Fig. 6I, Supplementary Data Table 7a, Supplementary Information).

## Discussion

Microbiome studies have become vital tools in medicine, ecology and evolution. However, best practices for many aspects of marker gene studies of microbiomes continue to develop. In this study we focus on the effects of different methods for annotation of organelle rRNA sequences, and their potential to influence biological conclusions.

### Cryptic mitochondria bias microbiome analysis.

In comparing samples that contain different mitochondrial sequences (including many cross-species comparisons), we find that differences in the accuracy with which mitochondrial reads are identified by taxonomic annotation pipelines can impact apparent microbial relative abundances, as well as community properties like alpha and beta diversity.

In cases where only a single mitochondrial sequence is present in each sample, it may be easy to detect if mitochondrial annotation has failed, because no reads will be annotated as mitochondrial. Investigators could then take ad hoc steps to remove mitochondrially-derived sequences. However, there are several mechanisms by which multiple types of mitochondrially-derived sequences may be present in 16S rRNA gene samples. For example, if the tissues of dietary, parasitic, or epiphytic organisms are co-mingled with the focal organism in samples, it can result in diverse mitochondria that must be annotated. Additionally, some animals and many plants show considerable heteroplasmy [41] in which mitochondrial genome sequence varies within the same individual. Levels of intra-individual sequence divergence between mitochondria can be substantial (e.g. up to 23% divergence reported in lobster mitochondrial 12S rRNAs [42]). Transposition of mitochondrial DNA to the nucleus, which is common (e.g. in humans [43]) can generate nuclear mitochondrial sequences (NUMTs). These are known to confound eDNA studies, and may also be amplified in 16S rRNA gene studies.

If any of these mechanisms are in operation, it is possible to annotate some but not all mitochondrially-derived sequences, offering researchers a false sense of confidence that all sequences have been correctly identified. In light of the results presented in this manuscript, it appears common for one or more of these mechanisms to create situations where only some mitochondrially-derived sequences in a 16S rRNA are correctly annotated. The approaches described here provide additional security against distortions due to cryptic mitochondrially-derived sequences when any of these common situations occur.

### Mitochondrial removal method interacts with study design to potentially alter biological conclusions.

In large datasets, the answers to biological questions often do not depend on the method used to remove mitochondria. However, we have identified 25 instances where use of our extended reference taxonomy resulted in altered biological conclusions. Specifically, when compared to the base references, 7 nominally significant results were revealed to be not significant at α = 0.05, and 18 nominally not significant results were revealed to be significant (Supplementary Data Table S7b, S7c). These examples include whether coral mucus, tissue and skeleton have distinct microbial communities as assessed by the Jaccard index; whether coral families differ in richness as assessed by Faith’s Phylogenetic diversity; whether milk storage silos significantly differ in Shannon diversity or Simpson’s evenness of their milk microbiome communities; and whether vertebrate classes (i.e. Mammalia vs. Reptilia) differ in gut microbiome evenness (Simpson’s evenness).

Alterations in biological conclusions based on mitochondrial removal method are most likely to occur in studies with few replicates and/or small effect sizes (e.g. when apparent p-values are close to 0.05). However, because of the need to adjust for multiple comparisons, small changes to any nominally significant p-value may nevertheless alter conclusions, depending on the experimental and analytical design of the study. We identify 53 cases in which the change in p-values between mitochondrial annotation methods was greater than ±0.05; these shifts in p-values may alter putative significance depending on the number of comparisons and method of multiple comparison correction. Finally, as many studies emphasize effect sizes and their 95% confidence intervals rather than significance per se, we also examined how mitochondrial annotation method impacted effect sizes. On average, the annotation method did not substantially change effect sizes (mean fold-change in effect size 0.998). However, we identify 5 cases where effect sizes changed by 2-fold or more, all of which were comparisons involving whether Peruvian ants differed in gut microbiome richness or evenness by habitat (Supplementary Data Table S6d).

We note that in order to standardize this analysis we ran each study through a common pipeline, so a difference in our analysis does not necessarily mean that the study conclusions themselves are suspect. For example, in several cases Deblur pipelines were used that resolve these issues, but our benchmarks warn that issues could have been encountered if, for example, DADA2 with neither additional filtering steps nor an extended mitochondrial reference taxonomy had been used.

### Effect of primer choice on mitochondrial removal.

We limited our analysis to the V4 hypervariable region of the 16S rRNA gene used in the EMP. However, many microbiome studies use more or different regions, which may offer more information to discriminate between bacterial and mitochondrial sequence data. In a study examining the utility of PNA clamps, Lundberg et al. (2013) show that commonly used primers overlap the 16S rRNA gene of both mitochondria and free-living bacteria, yet how closely those gene sequences mirror each other depends on the specific hypervariable region [44]. In particular, V1-V2 and V7-V8 appear to have less overlap than V4 and may offer more discriminatory power. Deissová et al. (2023) further demonstrated that roughly 70% of sequences amplified with V4 primers applied to human biopsy samples were off-target, host-derived reads. When they instead used a modified set of 68F-338R (V1-V2M) primers, the proportion of off-target reads was reduced to nearly zero [45]. One benefit of the bioinformatic approach identified here is that amplification of off-target sequences can be addressed *in-silico*, including retrospectively to standardized meta-analyses of already-collected microbiome data.

### Present benefits and future opportunities for improved mitochondrial annotations.

Cross-species microbiome comparisons, such as the GCMP, the Sponge Microbiome Project, and Song et al., often must identify many host species in the field during sample collection. In cases where this is challenging, correctly annotated mitochondrial sequences may offer clues. For example, the mitochondrial reads in the 16S rRNA gene amplicon data of the GCMP conflicted with the initial field identifications of several coral samples by divers. The identification of the coral species was subsequently revised based on the combined evidence provided by this molecular data and reexamination of sample photographs [11].

Laboratories may also be able to use analysis of mitochondrial sequences to detect and identify sources of contamination. For example, both human and bird mitochondrial sequences were detected in a small number of GCMP samples when unknown sequences were queried with BLAST (Supplementary Data Table S3), suggesting some samples that might be contaminated with non-host DNA during sampling or sequencing, and could be excluded from analysis.

### Recommendations

Our results suggest several actionable steps that can be taken for cross-species microbiome comparisons. First, researchers should be aware that high proportions of unknown sequences may be attributable (among other causes) to cryptic organelle rRNA sequences, both of the host organism, and any dietary or symbiotic eukaryotes. Second, by supplementing standard reference taxonomies with diverse mitochondrial sequences, as described here, researchers can in many cases greatly improve annotation of cryptic organelle sequences. Third, if such additions are not used, a positive filter against known rRNA sequences can remove divergent organelle sequences (as well as the sequencing artifacts that such positive filters were designed to exclude). Fourth, users of online repositories of marker gene data, including Qiita [46], should take care to check whether either a positive filter or an extended reference taxonomy has been applied. Fifth, cross-species comparisons of microbiome diversity should carefully implement all of these precautions, since the relative abundance of cryptic mitochondrial reads can vary across species. Sixth, using CoDA methods to detect differentially abundant taxa or changes in beta-diversity can avoid distortions to effect size and significance in the face of contamination by organelle rDNA. Finally, curators of taxonomic reference databases should take special efforts to include diverse mitochondrial and chloroplast sequences, as well as nuclear sequences derived from them (i.e. NUMTs homologous to mitochondrial rRNA genes) and recognize that diversity in these organelle sequences can be just as important as diversity within bacterial groups for correct annotation of amplicon data.

## Conclusion

We provide a simple method and supporting tutorials to supplement the commonly-used SILVA database with diverse mitochondrial sequences, and show that doing so improves annotation of cryptic mitochondrial reads, which in turn can yield more accurate biological conclusions. Consistent mitochondrial annotations will both help prevent bias in microbiome analyses, and can also provide important contextual information about studies, such as the presence of contamination from non-target samples.

## Supplementary Material

Supplemental_Results_Sonett_et_al_ISMEJ-comms_2024_Final_ycae114

## Data Availability

The 16S rRNA datasets analysed in the current study are publicly available in QIITA (qiita.ucsd.edu) under the following artifact ids: 2457, 2979, 3198, 3532, 3533, 3534, 3536, 3537, 3538, 31543, 54385, 54434, 54503, 54504, 54587, 55205, 56221, 82947. Mock communities analysed in the current study are available on GitHub, https://github.com/caporaso-lab/mockrobiota/blob/b7a161a5f3648be789cde9b88159438cde9689d9/inventory.tsv (ids 12–16 and 18–22). The extended taxonomies generated in this project are available in the Zenodo repository, https://doi.org/10.5281/zenodo.10251912. Project code and tutorials are available on GitHub, https://github.com/zaneveld/organelle_removal.
