# Supplementary material for: Organelles in the ointment: improved detection of cryptic mitochondrial reads resolves many unknown sequences in cross-species microbiome analyses
Source: ISME Commun. 2024 Sep 24;4(1):ycae114. doi: 10.1093/ismeco/ycae114 (PMC11631352; doi:10.1093/ismeco/ycae114)
Supplement: Supplemental_Results_Sonett_et_al_ISMEJ-comms_2024_Final_ycae114 [file supplemental_results_sonett_et_al_ismej-comms_2024_final_ycae114.pdf]

Supplemental Information for "The Organelle in the Ointment: improved detection of cryptic mitochondrial reads resolves many unknown sequences in cross-species microbiome comparisons"

Dylan Sonett[1], Tanya Brown[2], Johan Bengtsson-Palme[3,4,5], Jacqueline L. Padilla-Gamiño[6], Jesse R. Zaneveld[2#]

[1] *School of Pharmacy, University of Washington, Seattle, Washington, USA*

[2] *University of Washington, Bothell, School of Science, Technology, Engineering, and Mathematics, Division of Biological Sciences, Bothell, WA, USA*

[3] *Division of Systems and Synthetic Biology, Department of Life Sciences, Chalmers University of Technology, Gothenburg, Sweden*

[4] *Department of Infectious Diseases, Institute of Biomedicine, Sahlgrenska Academy, University of Gothenburg, Gothenburg, Sweden*

[5] *Centre for Antibiotic Resistance Research (CARe) at the University of Gothenburg, Gothenburg, Sweden*

[6] *University of Washington, School of Aquatic and Fisheries Sciences, Seattle, WA, USA*

[#]Corresponding author: Jesse Zaneveld, zaneveld@uw.edu

This PDF file includes:

Supplementary Results
Supplementary Methods
Supplementary Figure S1
Supplementary Figure S2
List of Supplementary Data Tables
List of Supplementary Data Files

Supplementary Results

A positive filter against known 16S rRNA sequences also prevents mitochondrial contamination. The default Deblur pipeline implemented in QIIME 2 includes a 'positive filtering' step. In this step, sequences are filtered against the Greengenes 88% OTU reference taxonomy. Those that do not fall within a 65% sequence identity threshold and 50% coverage threshold to this reference database are removed. Thus this positive filtering step demands that sequences broadly resemble known 16S rRNA sequences of free-living bacteria or archaea, or reference organelle sequences present in Greengenes. The threshold was selected to incorporate the range of known variation in bacterial and archaeal 16S rRNA sequences across phyla. If under-annotated mitochondrial reads are divergent, then this step may explain the better performance of deblur vs. DADA2 with default settings. To test this, we denoised sequences while either adding a positive filter to DADA2 or suppressing the default positive filter used in deblur (**Fig. 5**) and then annotated the results. By manipulating the positive filtering step in this way, we traced differences in mitochondrial annotation between deblur and DADA2 to the positive filtering step used in the QIIME2 implementation of deblur.

The cryptic mitochondrial or chloroplast reads detected when using an extended rather than base taxonomy seem to overlap heavily with divergent sequences excluded by Deblur's positive filter. Adding an identical SortMeRNA[1] (version 2.0) positive filtering step as is used in Deblur to DADA2 effectively eliminates the differences in how DADA2 and Deblur respond to the extended taxonomies. This is likely because adding the SortMeRNA positive filter to the DADA2 workflow causes cryptic mitochondrial reads to be filtered out, meaning that the extended taxonomy no longer changes the results much. Conversely, suppressing the positive filter from the Deblur workflow causes the extended taxonomies to matter much more than they otherwise would (**Fig. 5**). However, even with a positive filter, the extended taxonomies seem to influence mitochondrial annotations in some samples. For example, extended taxonomies reduced the number of samples with high levels of Unclassified sequences in the Song *et al.* dataset of diverse vertebrate microbiomes, even when a positive filter was present (**Fig. 5**).

Sequence artifacts simulated using shuffled sequences do not generate false positives using VSEARCH and the extended reference taxonomies. Another hypothesis we considered was that the extended taxonomies might cause increased false positive annotations of sequencing artifacts as mitochondria (perhaps due to increased incidental matches in nucleotide sequences). We tested this by shuffling the sequences from the GCMP coral dataset as well as sequences from the mock communities known to lack mitochondria. We expected the annotation for such scrambled sequences to be "Unassigned", since they should retain no non-random sequence similarity to known rRNA genes (sharing only their mononucleotide frequencies). After generating shuffled rRNA sequences, we then re-annotated these sequences using either base or extended taxonomies, and attributed any increase in mitochondrial annotations as evidence of either signal from mononucleotide frequencies (in the GCMP dataset only) or false positives (possible in either the mock or GCMP datasets equally). When using the VSEARCH classifier, which relies on sequence alignment, no sequences were annotated as mitochondria or chloroplasts, but rather were all Unassigned at the domain level (**Fig. S2a, S2c**).

When using the naive Bayes classifier, more shuffled sequences from the GCMP dataset were annotated as mitochondrial (4.5% vs. 0.001%) when using the extended rather than the base version of SILVA, with a corresponding change in 'Unassigned' annotations (**Fig. S2b, S2d**).

This was somewhat surprising — scrambling the sequences destroys all information other than nucleotide frequencies — and we expected any mitochondrial annotations in shuffled sequences to reflect increased false positives. However, we also tested the possibility that this machine learning method is using raw mononucleotide frequencies (which are not changed by scrambling) to identify mitochondrial reads.

If differences in mitochondrial annotation when using extended taxonomies were driven by false positives, we should expect them to also appear when the naive Bayes classifier is applied to mock community data known not to contain mitochondria. However, in that case, we saw no difference in the number of mitochondria annotated with base vs. extended taxonomic references using either VSEARCH or the naive Bayes classifier (**Fig. S2c, S2d**). This suggests that diversifying mitochondrial sequences in reference taxonomies does not increase false positive mitochondrial annotations. Interestingly, the extended taxonomies do change annotations of some previously unassigned non-mitochondrial sequences, reannotating sequences unassigned at the domain level to unclassified bacteria (**Fig. S2c**).

Compositional analysis is resistant to unidentified contaminants. Compositional data analysis (CoDA) methods account for differences in sequencing depth by modeling changes in the log ratio of pairs of microbial taxa or ASVs across samples. Removing features across an entire dataset increases the proportion of the remaining features evenly, leaving the log ratios between remaining features unaffected. We demonstrated this by running Analysis of Compositions of Microbiomes with Bias Correction (ANCOM-BC) on the GCMP dataset, as this was most heavily influenced by filtering pipeline. The ANCOM-BC results for differential abundance using family and tissue compartment in the GCMP dataset were identical regardless of the reference taxonomy used to filter the sequences. Thus, CODA methods protect against false positive identifications of differential abundance in non-mitochondrial ASVs. However, removing cryptic mitochondria is still advisable to avoid unnecessarily penalizing statistical power when correcting for multiple comparisons, and to avoid annotating any Unassigned reads that derive from mitochondria being reported as differentially abundant microbiome features.

PERMANOVA significance tests of Aitchison distances (**Fig. 6i**, **Supplementary Table 7a**) resulted in identical effect sizes when comparing unrarefied data filtered with the base or extended versions of the Silva taxonomy. Many p-values were also identical, and the slight variations in those that were not could be due either to the nature of the PERMANOVA test or alterations of the total sample number of features in each dataset. These changes are notably smaller than in other beta diversity metrics tested (weighted and unweighted UniFrac distances, Jaccard index, and Bray-Curtis dissimilarity), all of which had some alterations in both effect size and p-value. CoDA tools appear quite resilient to bias due to the presence of organelle sequences if they are unable to be removed.

Supplementary Methods

Testing changes to annotation of *in-silico* shuffled sequences. To further validate that extending the SILVA/Greengenes taxonomies did not increase false positive mitochondrial annotations, we generated shuffled versions of the GCMP data (high proportion of unknown sequences) and mock community data (zero unknown sequences). By calling the shuffle() method on each sequence in these datasets, true biological sequences were scrambled at the mono-nucleotide level. We reasoned that — aside from nucleotide composition — this procedure would destroy any biological signal in the sequences. Thus, any differential

annotation of these sequences would be attributable to either a) the minimal biological signal conveyed in nucleotide frequencies or b) false positive annotations.

Comparing ANCOMBC results. To test the effect of the extended reference taxonomies on compositional data analysis (CoDA) methods, non-rarefied feature tables from the GCMP coral dataset were filtered using the base and extended reference taxonomies and then analyzed with ANCOM-BC[2] in QIIME2, using the q2-composition ancombc method. The categories used for regression analysis were tissue compartment and family (formula option set to 'tissue_compartment+family'). Otherwise, default parameters were used.

Supplementary Figures

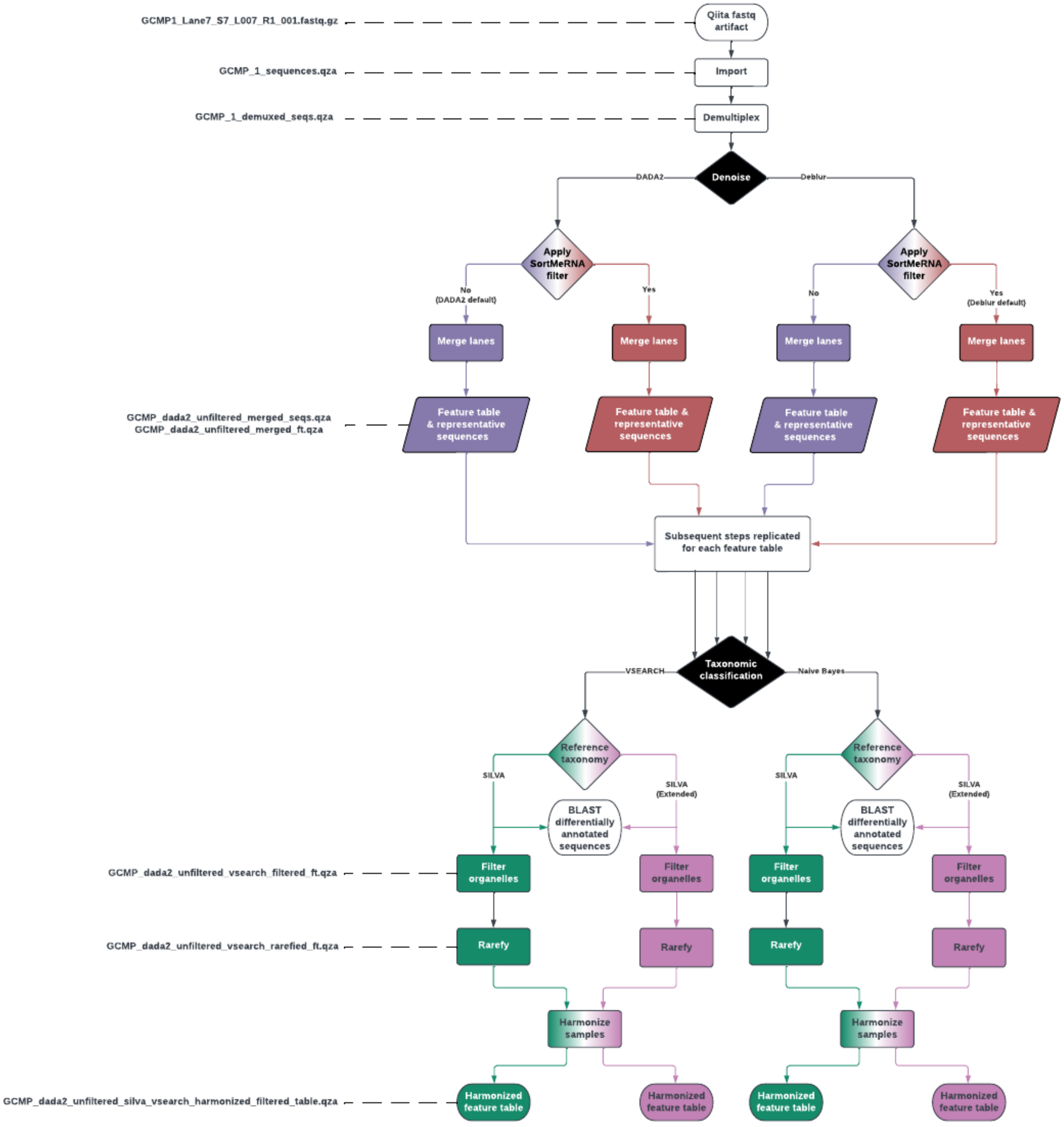

Figure S1. Workflow diagram for analyses in the study. Sequence files for each study in the analyses were downloaded from QIITA (6/7 studies) or mockrobiota (mock community samples). They were then denoised using either deblur or DADA2, with or without a positive filtering step against known sequences (this is default for deblur but not DADA2; see text). For each of the resulting 28 feature tables were run through taxonomic classification with either VSEARCH or a naive Bayes classifier from scikit-learn (as implemented in QIIME2), and with either SILVA or our extended SILVA reference taxonomy, resulting in 112 feature tables. Sequences identified as deriving from organelles were removed, each sample was rarefied to 1000 sequences per sample (discarding samples with fewer than 1000 reads). Finally, to enable fair comparison between rarefied base and rarefied extended taxonomies, only the intersection

of samples from these tables was analyzed. Additionally, all the above steps were repeated on Greengenes 13_8.

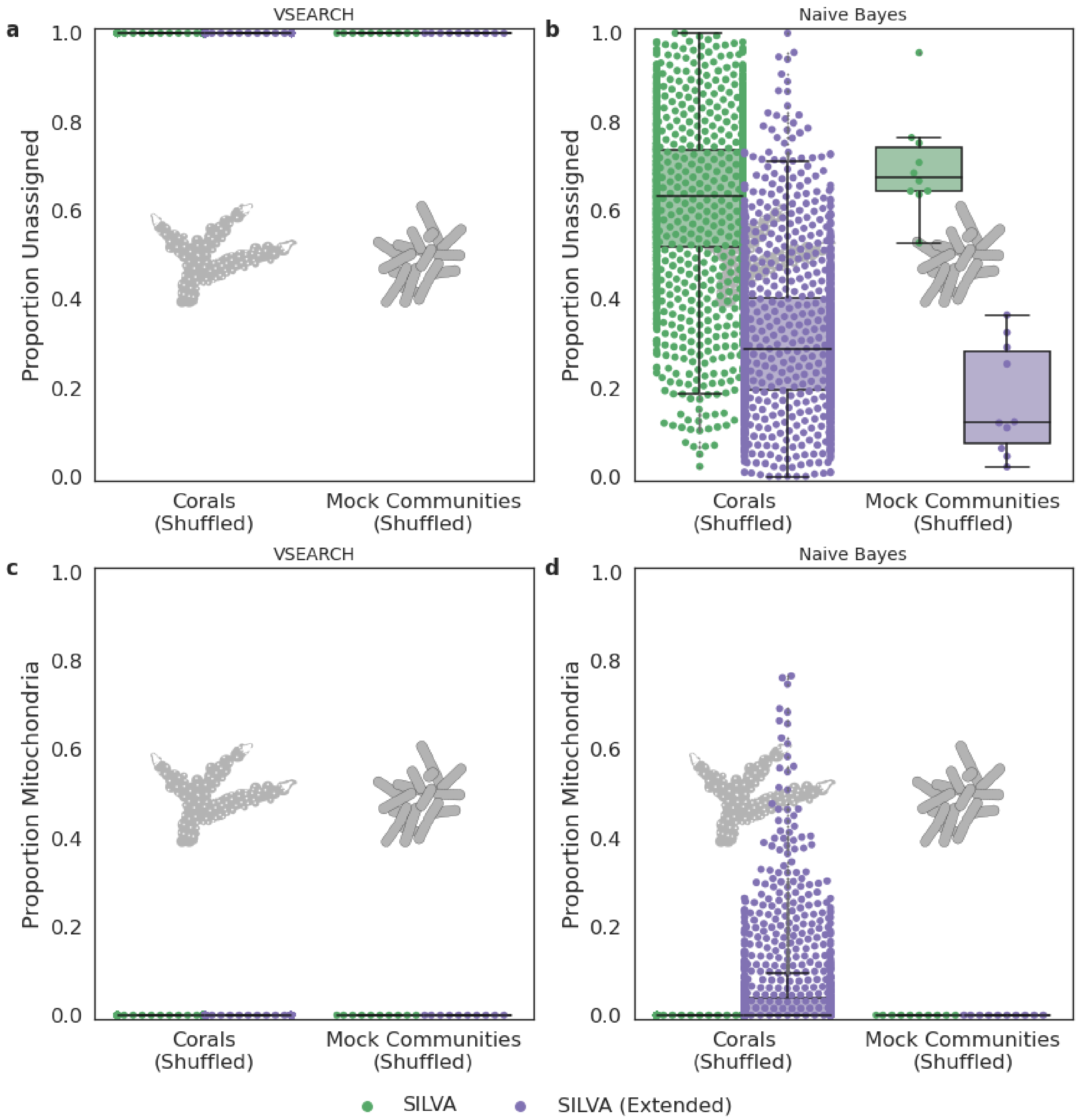

Figure S2. Extended reference databases do not increase false positive annotations of simulated sequencing artifacts. To test whether sequencing artifacts not removed by denoising might be classified as mitochondria when using extended reference taxonomies, we shuffled sequences of the GCMP (expected to contain mitochondria) and mock datasets (expected to contain no mitochondria), and used the VSEARCH (**a**, **c**) or naive Bayes (**b**, **d**) classifier to assign taxonomic annotations according the the base SILVA reference taxonomy (green). This procedure was repeated for the extended taxonomy (purple), with the distribution of “Mitochondria” annotations plotted on the y-axis. Using the different reference taxonomies,

the VSEARCH classifier showed no change in annotations of mitochondria. However, the naive Bayesian classifier annotated substantially fewer sequences as ‘Unassigned’ in each shuffled dataset, driven by an increase in Bacteria annotations (with no assignment at the phylum level) in both datasets, and an increase in Mitochondria annotations in the GCMP.

List of Supplementary Data Tables

Supplementary Data Table S1. Metadata for all studies in the analysis. **a.** Metadata for Pollock *et al*., the Global Coral Microbiome Project **b.** Metadata for Thomas *et al*., the Sponge Microbiome Project **c.** Metadata for Yatsunenko *et al*., study of human gut microbiomes. **d.** Metadata for Kable *et al*., study of bovine milk microbiomes **e.** Metadata for Sanders *et al*., study of Peruvian ant microbiomes. **f.** Metadata for Song *et al*., meta-analysis of vertebrate microbiomes

Supplementary Data Table S2. Effects of adding mitochondrial sequence diversity to taxonomic references. **a.** Proportion and absolute numbers of reads annotated as Unknown, Mitochondria or Chloroplast. Reports a summary of annotations for all combinations of denoising algorithm, base or extended reference taxonomies, and classification methods by sample. **b.** Five number statistical summary of the proportion or absolute number of reads annotated as Unknown, mitochondria or chloroplast by source study. This is a summary of the data in S2a, giving the minimum, first quartile, median, third quartile and maximum. **c.** Mann-Whitney U tests of proportions of samples annotated as ‘Unassigned’, ‘Mitochondria’, or ‘Chloroplast’ comparing base reference taxonomies to their extended counterparts.

Supplementary Data Table S3. Results of BLAST searches for top 1000 most abundant Unknown sequences identified in the Global Coral Microbiome Project dataset

Supplementary Data Table S4. Counts of organelle sequences in Greengenes 13_8 and SILVA 138 before and after supplementation with additional sequences.

Supplementary Data Table S5. Counts of sequences across all studies which were annotated differently by base SILVA 138 and SILVA (Extended) using VSEARCH. **a.** Domain-level reannotations. **b.** Phylum-level reannotations. **c.** Class-level reannotations. **d.** Order-level reannotations. **e.** Family-level reannotations. **f.** Genus-level reannotations. **g.** Species-level reannotations.

Supplementary Data Table S6. Results of negative control analysis testing for annotation of mitochondria in shuffled GCMP sequences using base or reference taxonomies.

Supplementary Data Table S7. Impact of mitochondrial annotation reference on alpha and beta diversity test statistics for **a.** all tests examined, **b.** tests that shifted from $p < 0.05$ to $p > 0.05$ when using extended taxonomic references. **c.** tests that shifted from $p > 0.05$ to $p < 0.05$ when using extended taxonomic references. **d.** tests with a > 2-fold difference in effect size when using extended taxonomic references.

Supplementary Data Files

Supplementary Data File 1. Extended version of the SILVA 138 V4 FASTA file, containing original SILVA sequences and additional mitochondria and chloroplast sequences. Along with Supplementary Data File 2, this allows for annotating the taxonomy of 16S rRNA gene amplicon

sequences (using QIIME2 or other microbiome software packages) according to the ‘extended’ taxonomies described in this manuscript.

Supplementary Data File 2. Extended version of the SILVA 138 V4 taxonomy file, mapping both original and extended sequences from the FASTA file to taxonomic annotations. Along with Supplementary Data File 1, this allows for annotating the taxonomy of 16S rRNA gene amplicon sequences (using QIIME2 or other microbiome software packages) according to the ‘extended’ taxonomies described in this manuscript.

Supplementary References

1. Kopylova, E., Noé, L. & Touzet, H. SortMeRNA: fast and accurate filtering of ribosomal RNAs in metatranscriptomic data. *Bioinformatics* **28**, 3211–3217 (2012).
2. Lin, H. & Peddada, S. D. Analysis of compositions of microbiomes with bias correction. *Nat. Commun.* **11**, 3514 (2020).
